# A method for large-scale implantation of 3D microdevice ensembles into brain and soft tissue

**DOI:** 10.1038/s41378-020-00210-5

**Published:** 2020-11-16

**Authors:** Stefan A. Sigurdsson, Zeyang Yu, Joonhee Lee, Arto Nurmikko

**Affiliations:** 1 School of Engineering, Brown University, Providence, RI 02912 USA; 2 Department of Neurology, Massachusetts General Hospital, Boston, MA 02114 USA; 3 Department of Physics and Astronomy, West Virginia University, Morgantown, WV 26506 USA; 4 Department of Neuroscience, West Virginia University, Morgantown, WV 26506 USA

**Keywords:** Engineering, Nanoscience and technology

## Abstract

Wireless networks of implantable electronic sensors and actuators at the microscale (sub-mm) level are being explored for monitoring and modulation of physiological activity for medical diagnostics and therapeutic purposes. Beyond the requirement of integrating multiple electronic or chemical functions within small device volumes, a key challenge is the development of high-throughput methods for the implantation of large numbers of microdevices into soft tissues with minimal damage. To that end, we have developed a method for high-throughput implantation of ~100–200 µm size devices, which are here simulated by proxy microparticle ensembles. While generally applicable to subdermal tissue, our main focus and experimental testbed is the implantation of microparticles into the brain. The method deploys a scalable delivery tool composed of a 2-dimensional array of polyethylene glycol-tipped microneedles that confine the microparticle payloads. Upon dissolution of the bioresorbable polyethylene glycol, the supporting array structure is retrieved, and the microparticles remain embedded in the tissue, distributed spatially and geometrically according to the design of the microfabricated delivery tool. We first evaluated the method in an agarose testbed in terms of spatial precision and throughput for up to 1000 passive spherical and planar microparticles acting as proxy devices. We then performed the same evaluations by implanting particles into the rat cortex under acute conditions and assessed the tissue injury produced by our method of implantation under chronic conditions.

## Neural interfaces: A high-throughput method for implanting microdevices

A low-injury, high-throughput technique to embed neural interfaces into brain tissue brings the idea of a ‘smart body’ closer to home. While some research groups focus on miniaturizing sensors and bio-actuators to implantable sizes, Brown University’s Stefan Sigurdsson and a team of US researchers have developed a technique to embed these devices into cortical tissue. In their method, microparticles (used in lieu of functional implants) are suspended in super-thin needles of polyethylene glycol, which are driven into the brain at high speed to minimize tissue damage. The polyethylene glycol is absorbed into the brain and the injection apparatus is removed, leaving the implants behind. The method can be scaled for rapid implantation of high numbers of devices and could see use in the development of human-computer integrations.

## Introduction

One vision of next-generation electrical and chemical in vivo biointerfaces is the idea of an ensemble of implanted active microscale devices forming a smart body-internal wireless network for sensing and stimulation of the underlying biological circuits. One actively pursued concept for developing large-scale neural interfaces, including efforts in our laboratories, envisions ensembles of wireless, autonomous microdevices spatially distributed in the brain^1–4^.

Concurrent with active microdevice development, we are presented with the question of how to implant these devices in a safe, scalable, high-throughput manner. To our knowledge, there are currently no reported methods for large-scale implantation of microscale devices. In this paper, we report the development of a means to implant large numbers of ~100–200 µm scaledevices. The method of implantation employs an array of microneedles carrying ensembles of devices as payload, with a bioresorbable polymer, polyethylene glycol (PEG), enabling their release upon implantation into tissue. The devices are constrained within PEG constructs that are shape-optimized for penetration and mounted onto the tips of a supporting array structure enabling parallel implantation of potentially large numbers of microdevices.

Throughout development of the method, we used microparticles in the form of spherical polymer microspheres and passive planar silicon chiplets as proxies for real microdevices. Microfabricated arrays, each carrying up to a thousand such microparticles, were tested in an agarose model to assess the spatial distribution and precision of microparticle delivery in full three dimensions. We performed experiments in rats in which we implanted ensembles of microparticles into the cortex for the in vivo assessment of our method in terms of both spatial precision and resulting tissue injury. Here, we report the results of these experiments and discuss the critical issues involved in enabling effective large-scale implantation while minimizing the associated cortical injury.

## Background

In the early phases of research, we investigated different approaches to implantation of microparticles as candidates for further development. As a baseline, we first performed a number of experiments exploring implantation by injection from a hypodermic needle. We noted how various groups had described methods of syringe implantation for the delivery of probes and other materials into brain tissue^5–8^. We utilized a 27 gauge needle (410 µm outer diameter, 210 µm inner diameter) as the microparticle carrier, with its plunger running the length of the needle for ejection of single ~100–200 µm size particles that were individually loaded into the needle tip (see Fig. S1a). We implanted particles (~200 µm diameter) to 25 locations (one by one) in the rat cortex to a depth of 1.4 mm in a chronic experiment but found that all the particles eventually returned to the cortical surface after a week-long period in vivo (see Fig. S1b). The finding that particles could not be properly secured in the tissue, likely finding a return pathway caused by injury from the necessarily large outer diameter of the syringe needle, led us to reject this method. Ideally, the diameter of the implantation track should match the diameter of the device. Equally important for us, the method should be readily scalable to large numbers of implantation sites (thousands) throughout the target tissue.

For assessment of a second candidate method of implantation, we next built a small benchtop apparatus resembling a “gene-gun”^9,10^ that allowed the microparticles to be accelerated through a pressurized nozzle to a speed exceeding 100 m/s. This enabled ballistic implantation of ~200 µm size particles into the mouse cortex to a depth of up to 1 mm in an acute experiment, but the excessive collateral tissue injury we observed in this case (see Fig. S2) led us to reject this method as well.

As a third candidate method, we arrived at the idea of incorporating PEG as a key material component into a custom designed and microfabricated implantation tool. Bioresorbable, naturally body-dissolving PEG has been used to stiffen flexible probes to improve penetration into brain tissue^11–17^ for implantation of a single or a handful of probes in parallel. In our case, PEG acts as a carrier for the release of a payload of microdevices rather than as a material to strengthen flexible probes, and the method is compatible with high-throughput implantation into soft tissues. Below, we describe the implantation process in more detail, as well as the process of microfabrication of the implantation tool.

## Results

### PEG-mediated implantation

**Fig. 1:**
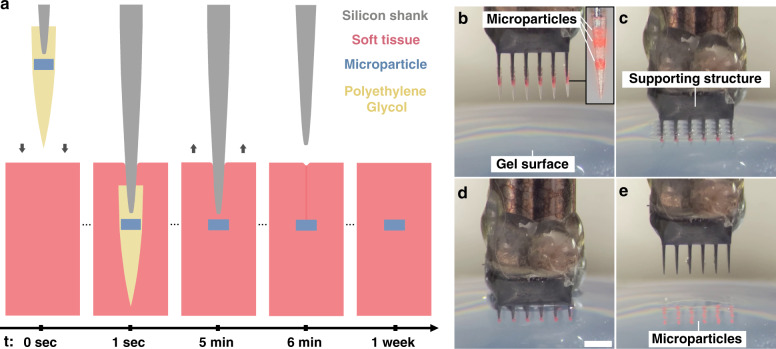
Overview of the implantation mechanics. **a** The polyethylene glycol (PEG)-mediated process by which microparticles (substitute microdevices) are implanted into tissue is depicted over time. Here, one particle is encased within a needle-shaped PEG construct. Upon immersion into tissue (aqueous environment), the PEG quickly dissolves. The particle is then left embedded in the tissue, and the supporting structure is retrieved minutes later. While depicted here for a single particle only, each PEG needle may carry several particles stacked vertically, and the process can be carried out in an array form for parallel implantation. **b–e** Implantation of a small 3D distribution of microparticles into agarose gel. **b** The implantation tool is positioned above the gel surface. The inset shows a magnified view of one PEG construct. **c** The implantation tool is slowly lowered into the gel. **d** Photograph taken immediately prior to retrieval of the supporting structure following dissolution of the PEG. **e** The supporting structure is slowly retrieved, leaving the microparticles embedded in the gel. Scale bar measures 1 mm

Figure 1 provides an overview of the process of PEG-mediated implantation, which is additionally displayed in video form in Video S1. In detail, each microparticle (a proxy for an eventual active device) was encapsulated within a needle-shaped PEG construct that constrained the microparticle to the tip of a supporting silicon shank. This configuration was replicated across each of the shanks of a larger supporting array structure forming what we refer to as the implantation tool (only one shank is illustrated in Fig. 1a for clarity; the array form is shown in Fig. 1b–e). Once assembled, the implantation tool could be driven into tissue (or a tissue mimic) by an insertion device, bringing the microparticle payloads to their target locations. Upon immersion into the aqueous environment, the PEG constructs would quickly dissolve, leaving the microparticles embedded in place and allowing the supporting array structure to be retrieved minutes later. For additional details of these procedures with respect to the relevant testbeds, refer to section “Testbed-dependent methods of implantation and assessment”. Note that the method was designed to be scalable to potentially hundreds of lateral locations across the target surface. Additionally, each PEG construct could contain up to 10 vertically separated microparticles, yielding a 3D distribution of particles within the implanted tissue. Specifically, for an array of 100 shanks, up to 1000 microparticles could be implanted in a single insertion step as per section “Unordered implantation of 3D microsphere ensembles into agarose”.

### Microfabrication of the implantation tool

**Fig. 2:**
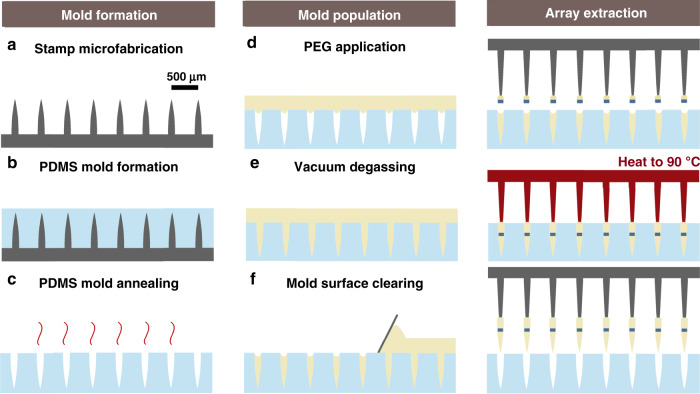
Illustration of the microfabrication process. The process by which implantation tools are fabricated proceeds as follows. First, an inverse poly-dimethyl sulfide mold of a microfabricated stamp is used to shape polyethylene glycol (PEG) into sharp needles. The mold is immersed in molten PEG at 90 °C in a vacuum for degassing to allow the PEG to conform to the shape of the mold. Following the molding of the PEG, microparticles are either placed directly into the molten PEG constructs or mounted separately onto the supporting array as displayed here. Finally, the needle-shaped PEG constructs are retrieved from the poly-dimethyl sulfide mold and mounted onto the supporting array structure by micromanipulators

The microfabrication process by which ensembles of microparticles are loaded into the supporting microarray frame is shown schematically in Fig. 2. The PEG constructs derive their needle-like shape from a positive stamp used for molding molten PEG. An important step in the process is the formation of this stamp, which we accomplished by two distinct processes, one that employs silicon microfabrication methods and the other a 3D nanoprinting technology. The 3D nanoprinting technology was explored as a more accessible and customizable option for stamp fabrication over the silicon-based process. The silicon process had the advantage in terms of throughput, however, as the nanoprinting process, given currently available moderate throughput and moderate cost equipment, would take several hours to produce even a relatively small stamp form factor (6 × 6 array). For details of both microfabrication processes, see section “Stamp microfabrication methods”.

The microfabricated stamps were used for the production of reusable poly-dimethyl sulfide (PDMS) molds, which in turn were used for molding PEG into the needle shape prescribed by each stamp (see section “Molding of PEG” for details of this process). Following the molding of the PEG (Fig. 2f), microparticle populations were either loaded directly into the molds or mounted onto the supporting array structure in a separate process by simple adhesion, as depicted in Fig. 2. The supporting array was then aligned with the PEG-populated mold by micromanipulators, and the PEG constructs were mounted onto the supporting array by temporarily rendering the PEG molten. While the PEG was in a molten state, the supporting shanks were able to slide into the constructs and attach as the PEG cooled down and solidified. The PEG constructs were then retrieved from the mold by raising the supporting array to complete the microfabrication sequence. Note that to consistently retrieve the molded PEG constructs from the molds without breaking at 100% yield, it was crucial that the molds be stiff and thoroughly annealed (see section “Molding of PEG”).

**Fig. 3:**
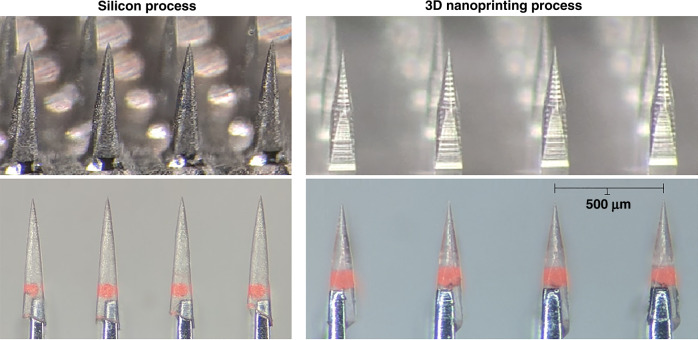
Comparison of stamp replication fidelity. Images of microfabricated stamps along with the corresponding molded polyethylene glycol constructs depicted for the two different stamp fabrication processes (silicon wet etching on the left, 3D nanoprinted structure on the right). The stamps are displayed in the top two panels, while the corresponding polyethylene glycol needles are displayed in the bottom two panels carrying fluorescent microspheres and mounted onto supporting array structures. The interneedle separation measures 500 µm in each case

Figure 3 provides a view of the positive stamps produced by the two microfabrication processes discussed in section “Stamp microfabrication methods”, in addition to the corresponding PEG constructs obtained through molding. The PEG constructs are seen here containing a single microparticle each and mounted onto the tips of supporting array structures that were themselves produced through a similar process as described for the silicon stamps (see section “Stamp microfabrication methods”). We performed SEM imaging of the stamps seen in Fig. 3 as well as their PEG counterparts to assess the degree to which the molding process accurately replicated the shape of the original stamp, specifically in terms of the needle tip size and surface roughness. For each sample, a measurement from 30 needles was obtained. For the silicon-based process, the stamp tip size measured 2.13 ± 0.47 µm (mean ± std), in contrast to the 2.37 ± 0.50 µm measured for the corresponding PEG needles. For the 3D nanoprinting process, the stamp tip size measured 1.25 ± 0.25 µm, with the respective PEG needle tips measuring 1.53 ± 0.32 µm. Surface roughness measured under 1 µm in all cases. The shape and surface roughness of penetrating probes have been shown to affect the extent of initial tissue injury caused by implantation^18,19^, so it is important that these parameters translate effectively from the microfabricated stamps to the PEG constructs.

### Spherical and planar microparticles as substitute microscale devices

**Fig. 4:**
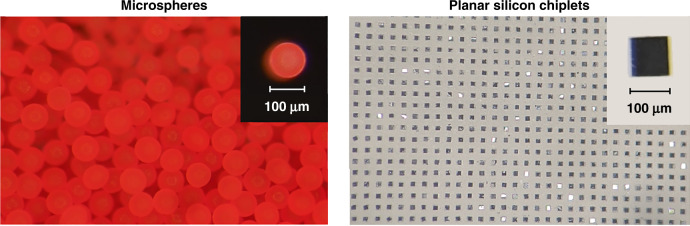
Substitute microdevices. The two types of substitute microdevices (microparticles) used in our experiments are displayed (fluorescent microspheres on the left, planar silicon chiplets on the right). The left-hand side displays an unordered collection of particles as received from the vendor, while the right-hand side displays particles organized by the use of a molded “waffle-pack”. The scale factor is shown in the inset for each type of microparticle

In lieu of active electronic microdevices or chemically infused microscale payloads, we chose two types of passive proxy particles to study the effects of different geometries and material choices: (a) spherical polyethylene and borosilicate microspheres of ~100 µm size purchased from Cospheric (UVPMS-BR-1.090, HCMS-BSGMS-FMB, Cospheric, CA, USA), and (b) planar silicon chiplets measuring 100 µm on the side and 50 µm-thick microfabricated through a combination of photolithography and dry etching (see section “Microfabrication of planar silicon chiplets”). We chose a size range near 100 µm recognizing ongoing efforts to create microelectronic and optoelectronic microscale active implants on that scale. We also performed experiments using 200 µm size microspheres, but as they produced qualitatively similar results as those obtained for the smaller sizes, we will not discuss them further. Figure 4 summarizes the two sets of microparticle shape and size used in our study.

To guide the process of loading and mounting the microparticles onto supporting arrays, PDMS molds in the form of a “waffle-pack” were produced (see section “Microfabrication of PDMS waffle-packs”) to enable organization of unsorted microparticles into a regularly spaced grid matching that of the stamps. Figure 4b displays sorted planar microparticles residing in such a mold, whereas Fig. 4a shows an unordered collection of microspheres as received from the vendor. The microparticles were organized using these molds by simple mechanical agitation of the particles placed on the mold surface (note that the molds must be thoroughly annealed for this purpose). The particles could then be mounted in parallel onto the supporting arrays by a small amount of PEG placed at the supporting shank tips. This allowed for high-throughput placement of both the microspheres and the planar chiplets into the PEG constructs at a rate of approximately a thousand microparticles for every few minutes of effort.

### Unordered implantation of 3D microsphere ensembles into agarose

**Fig. 5:**
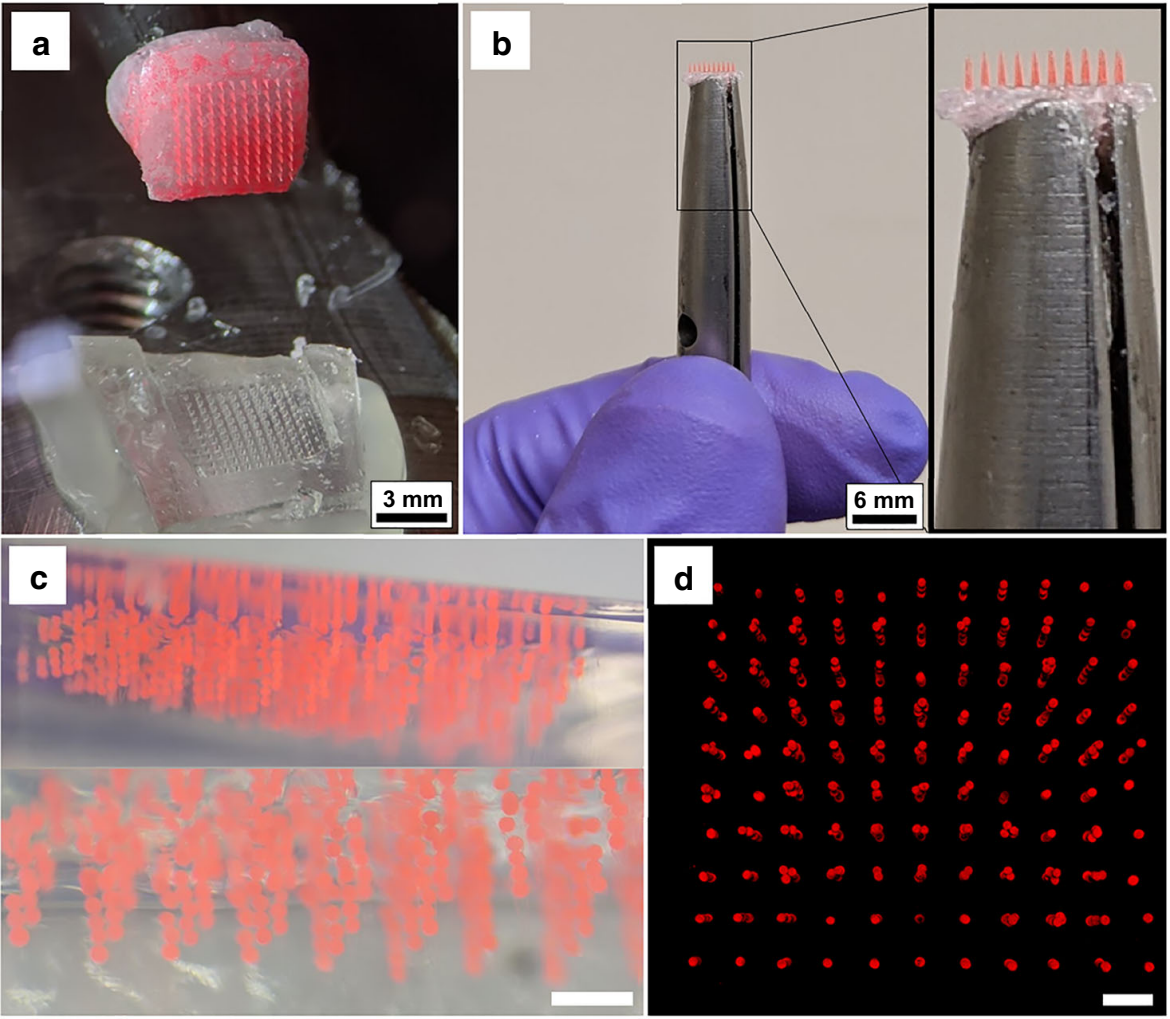
Unordered microparticle implantation. An unordered ensemble of ~600 microspheres was implanted into agarose. **a** Retrieval of the polyethylene glycol constructs from the mold. Note the omission of a supporting array structure, here replaced by a simple polyethylene glycol base. **b** Implantation tool held by hand for perspective. **c** The ensemble of microspheres implanted into agarose gel, which is displayed from a side view. **d** Reconstruction of the microsphere ensemble in agarose by confocal fluorescence microscopy displayed from a top view. Scale bars in **c** and **d** measure 500 µm

There are several ways by which groups of microparticles may be placed into the molded PEG constructs during preparation of the implantation tool. If one wishes to implant large numbers of microparticles (≥1000) across a 3-dimensional target volume, several microparticles can be placed within each PEG shank. A quick way of placing the microparticles into the molds is to simply pack the molds with particles to capacity before the application and vacuum degassing of the molten PEG. In this way, thousands of particles may be placed into a mold in a matter of minutes, although this approach results in a spatially unordered 3D distribution of microparticles after implantation. The results of this process are depicted in Fig. 5, in which an array composed of 10 × 11 = 110 PEG constructs was implanted into agarose, delivering a total of ~600 microspheres without the assistance of an additional supporting structure. Indeed, the use of a supporting array is not crucial to the process, and for some applications foregoing it may be possible depending on the physical characteristics of the tissue in question and the desired implantation depth. We found that the microspheres typically self-align along the vertical axis within each PEG construct, with a small spacing of 5–20 µm between each pair of microparticles, as seen in Fig. 5c. In some cases, surrendering control of the interparticle spacing and relying on this type of quasirandom self-assembly along the vertical axis may produce acceptable outcomes.

Following implantation, the agarose gel was imaged by confocal fluorescence microscopy for reconstruction of the microsphere ensemble within the gel. The 3D reconstruction is displayed in Fig. 5d and is available in video form in Video S2. Analyzing this reconstruction, the average number of microparticles within each of the vertical implantation columns was found to be 5.3 ± 2.5 (mean ± std). While we found that vertical self-alignment was prevalent, some aggregation was seen towards the top of the implantation columns, as seen in Fig. 5d. The needles of the stamp used for generation of the PEG constructs in this experiment tapered to ~200 µm width at their base, thus leaving some room for particles to be displaced from perfect vertical alignment near the base of the PEG constructs. We measured this horizontal displacement for each particle (from their respective implantation columns) of the ensemble and found that the average displacement (center-to-center) measured 28 µm, with 90% of particles displaced <55 µm. We believe this displacement, as well as the aggregation effect, can be significantly reduced with the use of a more optimized stamp shape. Nevertheless, these experiments demonstrated that the spatial distribution of microparticles is not significantly altered by the process of PEG dissolution within the agarose gel.

### Ordered implantation of planar silicon chiplet ensembles into agarose

**Fig. 6:**
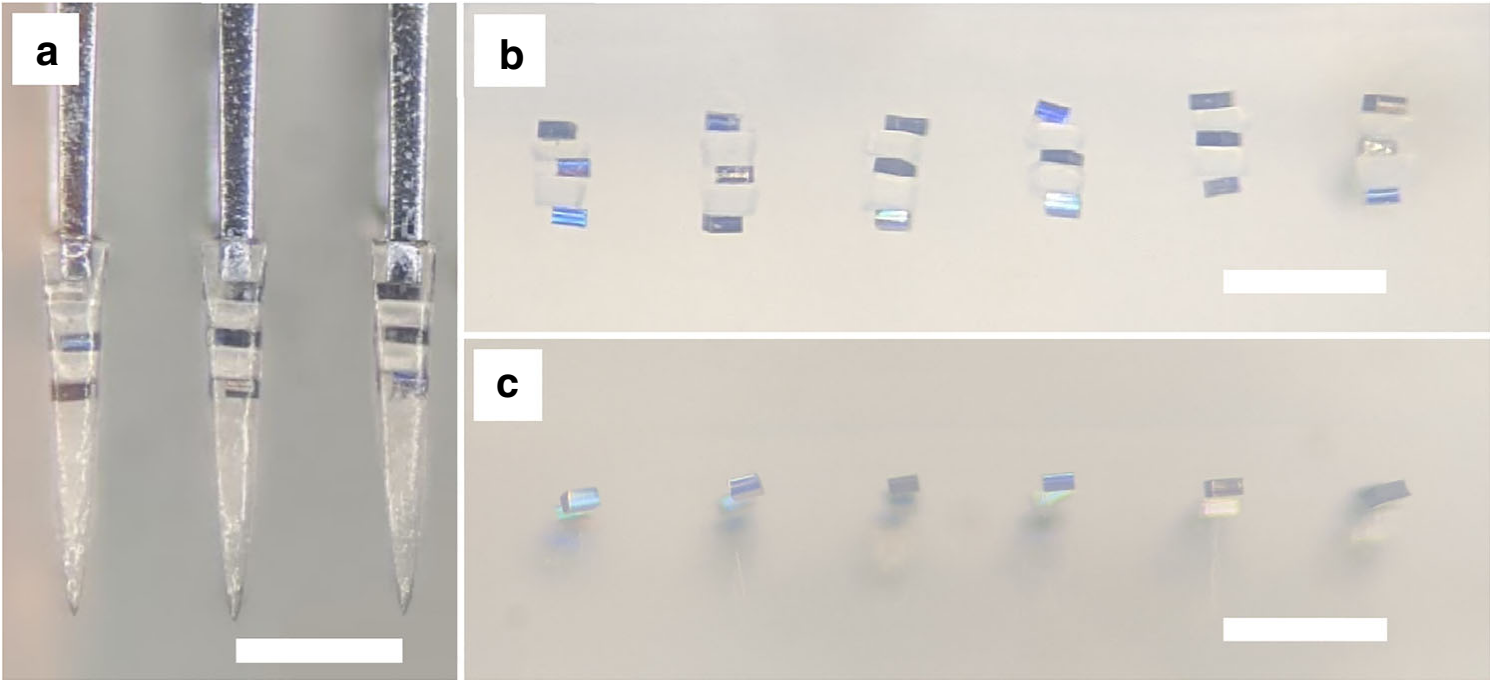
Ordered microparticle implantation. Ordered ensembles of planar silicon chiplets were implanted into agarose. **a** Multilayered ensemble of regularly spaced chiplets encased within PEG needles prior to implantation. **b** Side view of the same ensembles implanted into agarose. **c** Side-view of an implanted single-layer ensemble in agarose. Note the extent to which particle orientation and spacing is preserved on implantation. Scale bars measure 500 µm

Controlling not only the interparticle spacing but also the individual microparticle orientation may be important for active implants (e.g., antenna orientation for energy harvesting by inductive coupling^1^). Placement of microparticles in an ordered manner with a specific orientation into the PEG constructs introduces additional complexity, especially in terms of controlling intermicroparticle spacing for multilayered (3D) implant populations. Using the “waffle-pack” described in section “Spherical and planar microparticles as substitute microscale devices”, microparticles in the form of planar chiplets may be oriented and spaced appropriately in large numbers with minimal effort for high-throughput placement into the PEG constructs. For a single 2D layer of chiplets, the process is relatively straightforward. If several layers of chiplets are to be stacked within the same PEG construct, however, the process must be repeated several times with additional embedded spacer layers of correct thickness alternating with the chiplets. One convenient spacer material is silk fibroin. Microfabricated slabs of bioresorbable silk fibroin can endure the repeated heating of PEG to 90 °C while dissolving over time once implanted into tissue^20–22^. In this manner, the ordered implantation of large numbers of microparticles can be achieved, although the effort involved is proportional to the number of layers desired. As displayed in Fig. 6, the orientation of microparticles, as well as the intermicroparticle spacing, can be retained throughout the process of implantation into agarose. As an alternative to the layer-by-layer process, prearrangement of microparticles into columnar constructs for single-step placement into the molds could greatly reduce the effort involved. However, this process would need to be uniquely tailored to each kind of microparticle or microscale device.

### Acute in vivo experiments: Assessment of placement accuracy

**Fig. 7:**
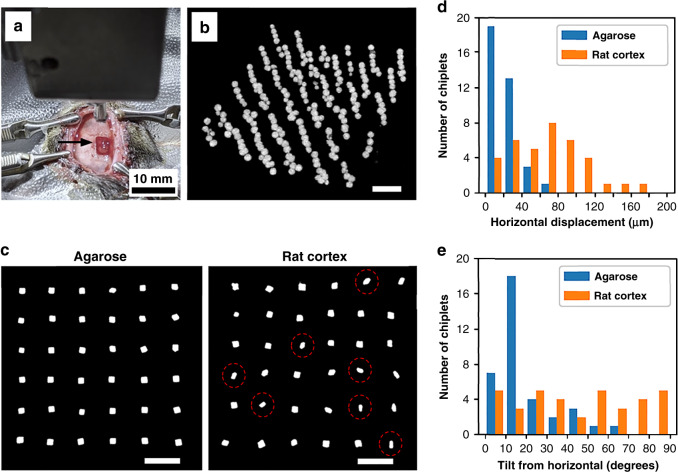
Implantation precision assessed in vivo. A single-layer ensemble of planar silicon chiplets and a multilayer ensemble of glass microspheres were acutely implanted into the rat cortex and imaged by a micro-CT scanner. **a** The experimental setup is displayed with the insertion device as well as the implantation tool above the target cortical location prior to implantation. The arrow indicates the exposed craniotomy. **b** Reconstruction of an intracortical 6 × 6 array 3D distribution of ~260 glass microspheres, viewed at an angle. **c** Reconstructions of two single-layer 6 × 6 array distributions of planar silicon chiplets displayed from a top view, one implanted into agarose and the other into the rat cortex. Some chiplets that have rotated towards a 90° tilt are circled in red. **d** and **e** Outcomes of the experiments in **c** were quantified. **d** Histogram showing the horizontal displacement of particles (20 µm bin size) from their respective vertical implantation columns (center-to-center) in agarose and in rat cortex. **e** Histogram showing the deviation of particles in terms of tilt in orientation (10° bin size) from the initial horizontal orientation in agarose and in rat cortex. Note the near uniform distribution of particle orientations in the rat cortex. Scale bars in **b** and **c** measure 500 µm

As the spatial distribution and orientation of the microparticles was adequately conserved on implantation into agarose, the question followed as to whether the same would apply to in vivo conditions, given the multiple biophysical forces that could affect the outcomes in living (brain) tissue. An acute rat model is a useful testbed even if the size of the animals’ head/brain limits the size of the overall implantable payload. We implanted a single-layer (2D) ensemble of 36 planar silicon chiplets into the rat cortex in an acute experiment. Separately, we also implanted a multilayer 3D ensemble of ~260 glass microspheres in a 6 × 6 array form factor into the rat cortex to study the implantation of 3D particle distributions (average 7.25 particles per PEG construct; the total number of particles was constrained by the head size of the rat model). The implantation process is depicted in Fig. 7a, with the insertion device and the implantation tool in view above the cortex. Surgical methods are described in section “Surgical methods”. We found that penetration into the cortex was consistent, with little or no dimpling of the cortical surface observed. In terms of overt tissue damage, we found that it was comparable to that of implantation of conventional microelectrode arrays immediately following implantation. Minimizing acute tissue damage is especially important, as we have found earlier how e.g. larger diameter hypodermic needles create damage tracks in brain tissue that act as channels for particle ejection (see Fig. S1). We note that in our chronic experiments, we did not observe particle ejection using the present method, in contrast to the hypodermic needle injection method.

The micro-CT scans obtained from the acute in vivo experiments involving both spherical (glass) microspheres and planar silicon chiplets were used to form 3D reconstructions of the particle ensembles (see Fig. 7b, c), which were analyzed similar to those described in section “Unordered implantation of 3D microsphere ensembles into agarose” above. While the microspheres might be expected to show minimal rotation due to their isotropic geometry, the chiplets possess large shape anisotropy whereby reorientation (tilt) might occur. Information was first gathered about the horizontal (in-plane) deviation of the chiplets from their respective vertical implantation columns (center-to-center). We then focused on the deviation of the chiplets in terms of tilt from the initial horizontal plane. The results are displayed in Fig. 7d, e in the form of histograms. Here, data from identical agarose experiments are overlaid to give a sense of the difference between the two experimental conditions. We found that chiplet positioning in the horizontal plane was more robust in agarose (mean 21 µm deviation) than in vivo (mean 67 µm deviation), perhaps largely due to swelling of the tissue, although both conditions produced satisfactory results for our needs.

As for deviations in chiplet orientation, i.e. tilt, the noticeable distribution of tilt angles in vivo with respect to that in agarose shows how biophysical forces in the living cortex could drive an initial (near) zero-tilt angle distribution towards a 90° tilt after implantation. While the driving forces at play in tilting the chiplets by 90° are unknown to us, there could be both intrinsic structural reasons (here cortical columns) and extrinsic forces (residue of implantation damage) that favor such an “edgewise” or vertical realignment of the chiplets. For active microelectronic devices, such reorientation must be taken into consideration when designing orientation-sensitive features such as antenna structures for energy harvesting. A major difference between the brain tissue environment and the agarose is that the tissue is dynamic. The tissue pulses with every heartbeat, expanding and contracting in a way that may influence the orientation of implanted devices. Investigation into the specific cause of the observed dynamic reorientation is the subject of current study, which aims to identify strategies by which device orientation may be preserved or otherwise controlled if so needed.

### Chronic in vivo experiments: Assessment of cortical injury

**Fig. 8:**
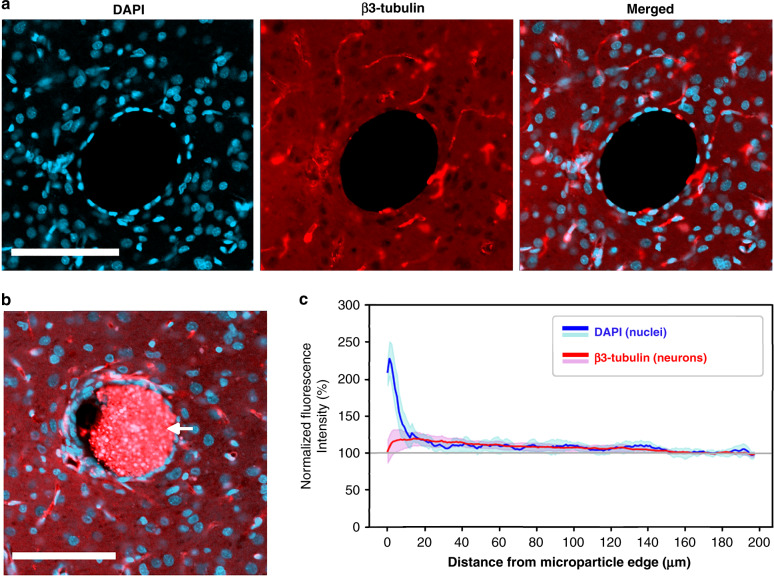
Assessment of chronic tissue injury. Characterization of the tissue reaction to microparticles implanted for 4 weeks in the rat cortex. **a** Representative view of a microparticle void and the surrounding tissue with cell nuclei labeled blue (DAPI) and neurons labeled red (β3-tubulin). **b** Example of a tissue section with a sliced microparticle still present (arrow). **c** Normalized DAPI and β3-tubulin fluorescence pixel intensities plotted as a function of distance (1 µm bins) from the microparticle edge. Each trace is the average of data collected from the vicinities of *n* = 30 microparticles across four rats. Shaded areas represent one standard deviation. Scale bars measure 100 µm

For assessment of the cortical injury produced by our system, we implanted unordered 3D microparticle ensembles into the cortex in four rats to a cortical depth of 800–1400 µm (by use of supporting arrays). Each ensemble totaled between 100 and 150 fluorescent polymer microspheres in a 5 × 6 array form factor. At 4 weeks postimplantation, the animals were perfused, and their brains were dissected. The brains were consecutively cryosectioned in the horizontal direction at a 20 µm slice thickness, and every third slice was then stained with β3-tubulin for labeling of neurons and with DAPI for labeling of cell nuclei (for details, see section “Immunohistology methods”). The slices were then imaged by confocal fluorescence microscopy, and the neuronal and nuclear presence around the implanted microparticles was characterized. In the vast majority of cases, the sliced fluorescent polymer microspheres were ejected from the tissue slices during the gentle treatment steps prior to imaging (see Fig. 8a, b), so to identify particle sites in the cortical slices, we looked for the presence of voids larger than 80 µm in diameter (the microspheres measured 90–110 µm in size). Voids of this size were not found in the healthy hemispheres of the subjects, while hundreds of such spherical voids were found across the affected hemispheres in a roughly 5 × 6 configuration. For any voids meeting the size criterion, we imaged and then calculated the normalized β3-tubulin and DAPI fluorescence intensities (per pixel) as a function of distance (1 µm bins) from the edge of the microparticle void. Normalization was performed with respect to the fluorescence intensity measured at a 160–180 µm distance from the microparticle voids. No identified voids measured larger than 110 µm, which is consistent with the size range of the implanted microspheres. Aggregate results are displayed in Fig. 8c for 30 microparticle voids across four rats imaged under standard conditions. As can be seen in the figure, we did not find a significant drop in β3-tubulin intensity in proximity to the microparticles, indicating that the proximal neuronal population was not significantly diminished by our implantation method, the PEG, or the chronic presence of the microparticles themselves. We did, however, find an increase in DAPI intensity near the microparticles, indicating the formation of an encapsulating layer of tissue extending 15–20 µm from the microparticle edge. As the neuronal decline, as well as the spatial extent of the encapsulating tissue, was well within normal limits found for other intracortical devices^23,24^ (see e.g. the neuronal decline described by Biran et al. ^23^), detailed immunological characterization of the encapsulating tissue was not deemed a priority and is left as the subject of future experiments.

We note that, in these chronic experiments, we used polymer microspheres (as opposed to glass or silicon particles) to enable effective cryosectioning of the cortical tissue. This means that detailed analysis of the spatial distribution of the implanted microparticles could not be performed by any imaging modalities available to us, so we could not thoroughly assess the spatial precision of particle delivery (or subsequent particle drift) under chronic conditions as we had done in the previous sections. During cryosectioning, however, we did observe a clear 5 × 6 array form factor of the implanted microparticle distribution, matching the form factor of the corresponding implantation tools, in addition to an appropriate ~600 µm vertical spread of the microparticle population. This indicates that particle drift postimplantation was limited and that chronic applications of implanted microscale devices would be supported by our method. Given this, a more thorough assessment of the extent of particle drift was not deemed an immediate priority and is left as the subject of future experiments.

## Discussion

The development of systems of implantable microscale devices for application in the body is the subject of ongoing research. While numerous groups have published efforts towards miniaturization of active devices, a question that remains unanswered is how these kinds of devices could be implanted into tissues in large numbers at high throughput. The method we have described here for this purpose is applicable to a variety of device geometries and system configurations for high-throughput implantation into the nervous system as well as generally into other soft tissues. As researchers look for methods to enable implantation of their devices, the microfabrication process we have developed could be used for incorporation of those devices into appropriate implantation tools. With those tools provided, the only component requiring operation at the hands of the researcher would be the micromanipulator-mounted inserter described in section “Testbed-dependent methods of implantation and assessment”. Alternatively, researchers could opt to produce their own customized implantation tools with relative ease via the microfabrication process we have described.

As demonstrated by our experimental results, the method of implantation presents a reliable means of delivering large ensembles of microparticles to precise locations in the cerebral cortex, a particularly challenging bioenvironment. Regarding tissue injury, we found no signs of neuronal loss around chronically implanted microparticles 4 weeks postimplantation. We did find signs of encapsulating tissue in the same experiments, although these extended only 15–20 µm from the surface of the microparticles. While a detailed analysis of the encapsulating tissue was not performed, our results indicate that the implantation of microparticles by our system produces chronic tissue injury comparable to that of conventional intracortical devices.

As discussed in section “Chronic in vivo experiments: Assessment of cortical injury”, we found that the implanted microparticles did not migrate substantially from their original locations under chronic in vivo conditions. Detailed quantification of this migration was not possible, however, nor could we quantify the change in particle orientation under chronic conditions. This, in addition to the challenge of the safe in vivo extraction of implanted microparticles, is the subject of current work aimed at exploring the full device lifecycle in chronically implanted animals.

We note that while we focused on implantation of microscale devices, the implantation of arrays of flexible electrodes (or similar physiological probes) is in principle also supported by our method. Further, by way of comparing another advanced implantation approach for embedding microprobes into the brain cortex, we note the system being developed by Neuralink^25^. The reported rate of implantation for that system amounts to six separate implantation sites across the cortex per minute, although they implant only a single probe at a time. By tailoring our approach, implantation tools of 100 shanks each could conceivably be implanted in a similar manner for a throughput exceeding that reported so far by two orders of magnitude.

In conclusion, we have developed a means of implanting large numbers of microscale devices into tissue at high throughput. We performed in vivo experiments in the cerebral cortex to assess our method in terms of spatial precision and induced chronic tissue injury. The results of our experiments indicate that the method is suitable for the large-scale implantation of microscale devices, justifying further efforts towards the development of various microdevice-based technologies.

## Materials and methods

### Testbed-dependent methods of implantation and assessment

The implantation system we developed was assessed by studying microparticles implanted into agarose brain phantoms (0.6% w/w in deionized water) and the in vivo rat cortex.

The agarose, which mimics the mechanical features of the brain^26^, served to provide a baseline for study of the implantation mechanics in a relatively controlled and static environment. Insertion of implantation tools into agarose gels was accordingly performed in a controlled manner, using a micromanipulator to lower the implantation tools into the gels at a speed of ~0.5–1.0 mm/s. Upon immersion into the agarose, the PEG constructs would completely dissolve within the span of one minute, as identified by visual inspection. Providing some margin to allow the microparticles to become securely embedded in the gels, the supporting structures were generally retrieved 3–5 min following insertion. Minimal particle displacement could be observed by microscopy throughout this retrieval process.

The rat cortex served as a useful testbed for in vivo assessment of the implantation method. In contrast to the slow insertion speeds used in agarose, the implantation tools were inserted at high speed into the rat cortex to prevent the dimpling of the cortical surface that would otherwise occur. Note that high-speed implantation of microelectrode arrays (of a similar shape as our implantation tools) has been reported to reduce dimpling of the cortex on implantation as well as the resulting tissue injury^27–29^. As our implantation tools could not rest on (or near) the cortical surface prior to insertion due to the fast dissolution rate of the PEG constructs, conventional pneumatic insertion devices with a 1.5 mm stroke length could not be used. Instead, we purchased a solenoid actuator (ac120220v306, Uxcell, Hong Kong, China) with a 10 mm stroke length for insertion of our implantation tools, which could then be comfortably positioned 10 mm above the cortical surface prior to implantation. The solenoid actuator, when powered at 120 VAC, would drive the implantation tools into the cortex at a speed of ~5 m/s as measured by a high-speed camera, which proved adequate for our needs and is in the range of speeds enabled by conventional pneumatic inserters of microelectrode arrays^27^. Following their insertion, the implantation tools were left within the cortex for 5 min to allow the microparticles to become securely embedded within the tissue. The 5-min duration was found to produce adequate results, although the effects of altering this duration were not thoroughly investigated.

Given the transparency of agarose, the implanted fluorescent microspheres could be imaged by confocal fluorescence microscopy (FV3000, Olympus, Tokyo, Japan) for spatial reconstruction of the embedded particle distributions. For particles implanted into the rat cortex, imaging was performed by a micro-CT scanner (microCT 40, Scanco Medical, PA, USA) to overcome the optical opacity of brain tissue. Note that this imaging modality was incompatible with our polymer microspheres, however. In either case, 3D reconstructions of the implanted microparticle ensembles could be generated at a resolution of just a few micrometers to study the spatial precision of our implantation system.

### Stamp microfabrication methods

Stamps were produced for molding by two distinct processes. One process is silicon based, involving mechanical dicing and wet etching of bulk single-crystal silicon to produce the tapered needle shape appropriate for penetration into cortical tissue. In short, a 2.25 mm-thick silicon wafer was diced into several shank arrays, with each shank measuring 225 µm by width and 1500 µm by height at a 500 µm separation between shanks (center-to-center). The arrays were then wet-etched in a 1:19 ratio solution of 50% hydrofluoric acid and 70% nitric acid. The etching time was varied across the arrays to produce an assortment of subtly different shapes. Various groups have described the details of this process for the production of microelectrode arrays, such as for intracortical neural recording^30–33^.

The second process, to be described more fully in a later publication, takes advantage of 3D nanoprinting technology by which the stamps were formed through layer-by-layer polymerization of photoresist (Photonic Professional GT system, Nanoscribe GmbH, Karlsruhe, Germany). We produced arrays of needles using IP-S photoresist (Nanoscribe IP-S, Nanoscribe GmbH) at a 1 µm process resolution, with each needle measuring 125 µm by width and 550 µm by height at a 500 µm separation between needles (center-to-center). For additional details of this process, refer to the methods of Faraji Rad et al. ^34^

### Molding of PEG

Molds adopting the shape of the microfabricated stamps were formed using PDMS (Sylgard 184, Dow Corning, Michigan, USA), which was mixed at a 1:7 ratio of hardener to elastomer for adequate stiffness. Once degassed, the PDMS was cured at 50 °C for 6 h to form a mold around the selected stamp, and each mold was then annealed at 120 °C for 24 h after the stamp was retrieved.

For molding of the PEG into appropriate needle-shaped constructs, the PDMS molds were immersed in molten PEG, after which a vacuum oven was used for degassing of the PEG at 90 °C for 10 min, allowing the PEG to conform to the shape of the molds. PEG is rendered molten at ~65 °C, so an elevated process temperature is necessary. For the choice of large molecular-weight PEG (35 kDa; CAS 25322-68-3, Sigma Aldrich, Missouri, USA), the reader is referred to the discussion by Lecomte et al. ^11^

### Microfabrication of planar silicon chiplets

For the production of planar microparticles, a 50 µm-thick silicon wafer was first mounted onto a carrier wafer by a thin layer of thermal grease (AOS 340 WC, AOS Thermal Compounds, NJ, USA). An S1813 photoresist mask (Microposit S1813, Shipley Company, MA, USA) was then patterned onto the thin wafer by photolithographic methods (defining the square microparticle shape), and the wafer was etched by deep reactive ion etching (Omega LPX Rapier, SPTS Technologies, Newport, UK). The sectioned microparticles were then released in acetone and cleaned by an ultrasonic cleaner (Elmasonic P, Elma Schmidbauer GmbH, Singen, Germany).

### Microfabrication of PDMS waffle-packs

For the production of waffle packs, a stamp was first produced by patterning SU-8 photoresist (SU-8–2100, MicroChem Corporation, MA, USA) using photolithographic methods into a regularly spaced grid of ~100 × 100 × 100 µm^3^ blocks on a silicon wafer at a 500 µm interblock spacing (center-to-center). This wafer was then used for the production of PDMS molds per the methods described in section “Molding of PEG”. Note that the size of the SU-8 blocks was selected such that only a single planar microparticle could fit within each of the voids in the waffle-packs that were formed.

### Surgical methods

Evaluation of our in vivo implantation methods was carried out as follows. Male Long Evans rats (8 weeks old, obtained from Charles River Labs, MA, USA) were anesthetized by isoflurane (1–3% concentration in oxygen) and mounted onto a stereotaxic apparatus. A craniotomy measuring 4 × 6 mm^2^ was performed over one hemisphere between lambda and bregma, with the dura mater subsequently removed by sharp tweezers. Sterile saline was continuously applied to the cortical surface while the craniotomy was exposed. The implantation tool was secured over the exposed craniotomy, and the insertion device (described in section “Testbed-dependent methods of implantation and assessment”) was used to drive the array of microparticles into the cortex at high speed. Five minutes were then allowed to pass before the supporting array structure was slowly retracted, leaving the particles embedded in the tissue. Animal care and experiments were performed in accordance with the National Institutes of Health guidelines and approved by the Brown University Institutional Animal Care and Use Committee (protocol #1704000267).

### Immunohistology methods

For histological analysis, rats were terminally anesthetized with pentobarbital (intraperitoneal injection). Transcardial perfusion was performed with 0.1 M phosphate buffered saline (PBS), followed by 4% paraformaldehyde in 0.1 M phosphate buffer (PB). The brain was extracted and fixed in 4% paraformaldehyde in PB overnight and then cryoprotected in 30% sucrose solution (until the brain sunk in the solution). The brain was then frozen with dry ice and sectioned into 20 µm-thick horizontal slices using a microtome. Brain slices were then washed in PB (twice, 5 min each) and PBS (twice, 5 min each) and left for 1 h in blocking solution containing 10% goat serum (ab7481, Abcam, Cambridge, UK), 0.25% Triton-X 100 (Sigma Aldrich), and 0.1% Tween (Sigma Aldrich) in PBS. The slices were then stained overnight at 4 °C in blocking solution containing beta III tubulin (β3-tubulin) antibodies for labeling of neurons (1:100 ab201740 Anti-beta III tubulin antibodies conjugated with Alexa Fluor^®^ 594, Abcam). The slices were then washed in PBS (twice, 5 min each) and PB (twice, 5 min each) and mounted onto microscope slides with diamidino-2-phenylindole (DAPI) mounting medium for fluorescence. Finally, the slides were imaged by confocal fluorescence microscopy for evaluation (FV3000, Olympus).

## Supplementary information

supplementary video 1

supplementary video 2

Supplemental Material

## Data Availability

The datasets generated and analyzed in the current study are available from the corresponding author on reasonable request.
