# Supplementary material for: A method for large-scale implantation of 3D microdevice ensembles into brain and soft tissue
Source: Microsyst Nanoeng. 2020 Nov 16;6:97. doi: 10.1038/s41378-020-00210-5 (PMC8433454; doi:10.1038/s41378-020-00210-5)
Supplement: Supplementary file 3 — Supplemental Material [file 41378_2020_210_MOESM3_ESM.docx]

Supplementary Materials

A method for large scale implantation of 3D microdevice ensembles into brain and soft tissue

Stefan A. Sigurdsson^1*^, Zeyang Yu^2^, Joonhee Lee^3,4^, and Arto Nurmikko^1*^

^1^School of Engineering, Brown University, Providence, RI 02912 USA

^2^Department of Neurology, Massachusetts General Hospital, Boston, MA 02114 USA

^3^Department of Physics and Astronomy, ^4^Department of Neuroscience, West Virginia University, Morgantown, WV 26506 USA

* Corresponding author: [stefan_sigurdsson@brown.edu](mailto:stefan_sigurdsson@brown.edu); [arto_nurmikko@brown.edu](mailto:arto_nurmikko@brown.edu)

**
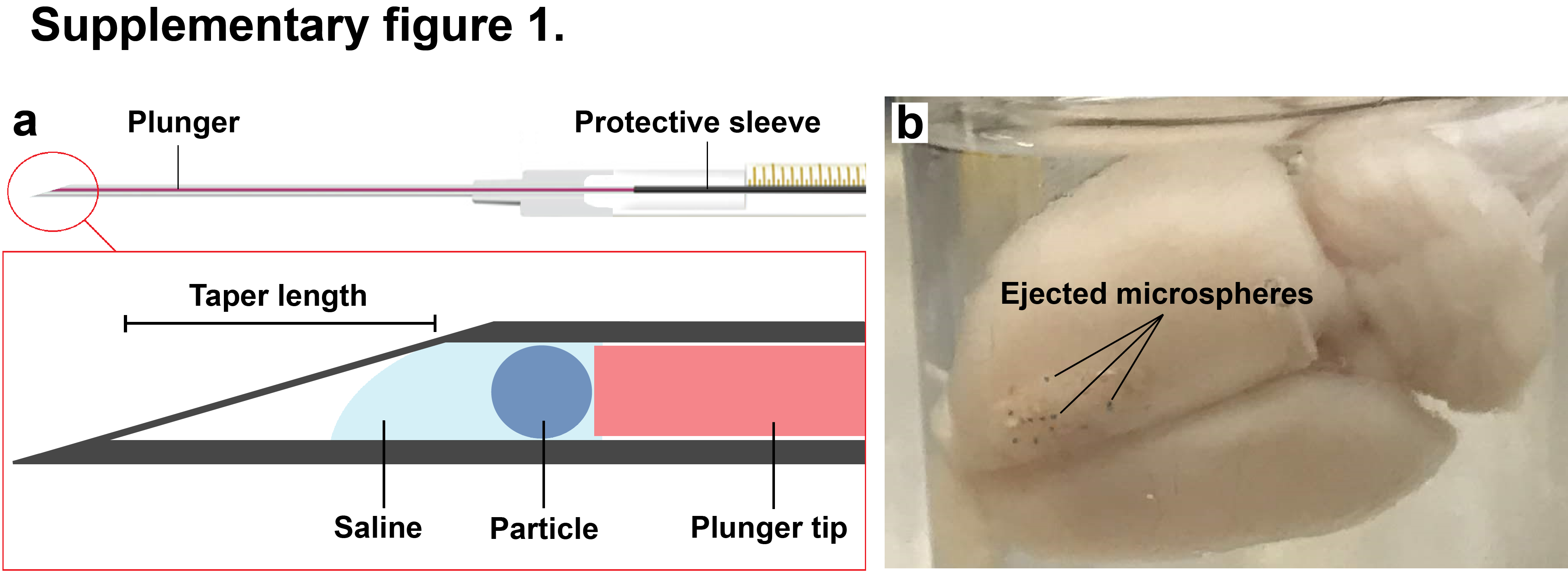
**

**Fig. S1.** Summary of syringe injection experiments. **a** Illustration of the syringe configuration prior to implantation of a single microparticle. With the syringe needle inserted into tissue to the target location, the plunger could be extended for ejection of the single particle into the tissue where it would remain embedded on retrieval of the syringe. **b** Extracted brain from a rat into which 25 microparticles were implanted by syringe injection in a survival experiment. Over the course of the 7 day experiment, all 25 particles were apparently ejected from the cortex. Some are seen scattered across the cortical surface here. Complete absence of particles within the cortex was verified via cryosectioning. Note that no particles were seen emerging from the tissue throughout the initial survival surgery.

**
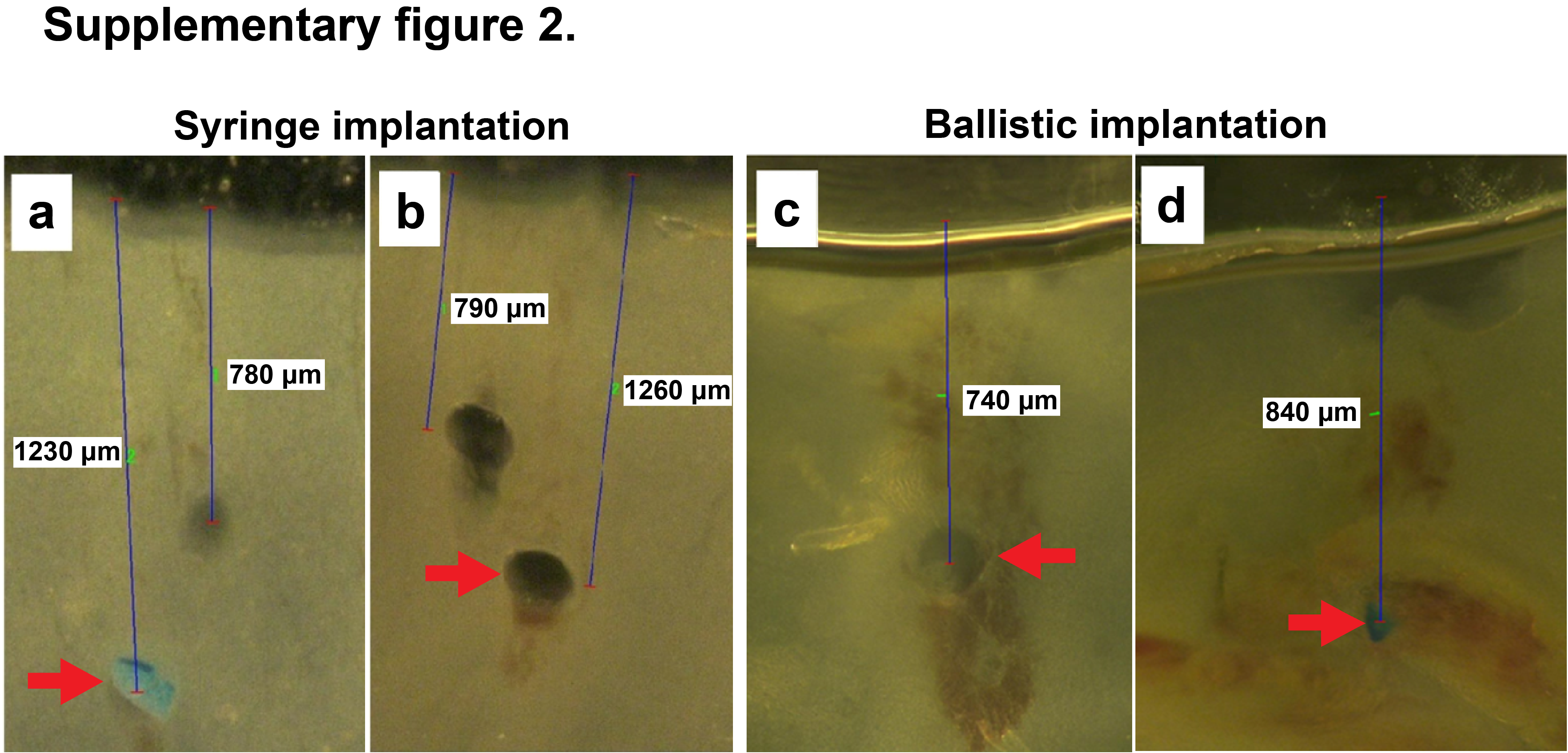
**

**Fig. S2.** Comparison of acute intracortical bleeding in coronal mouse tissue slices under white light illumination. Arrows point to microparticles, as well as voids left behind by microparticles. **a-b** Examples of bleeding around microspheres implanted by syringe injection. **c-d** Examples of bleeding around microspheres implanted ballistically. Note the excessive bleeding observed in this case, which we interpret as being the consequence of the large kinetic energy required for particles to penetrate to a cortical depth of ~1 mm.

**Video S1.** A small collection of microspheres is implanted into agarose gel for demonstration of the implantation process. The implantation tool is seen slowly inserted into the gel here, whereas *in vivo* the implantation tools are inserted at high speed to prevent dimpling of the cortical surface.

**Video S2.** The unordered ensemble of ~600 microspheres implanted into agarose that is displayed in figure 5d is shown rotating as a 3D reconstruction obtained by confocal fluorescence microscopy.
